# Elective flexible ureteroscopy with suction sheaths for infectious stones in prior UTI patients

**DOI:** 10.1002/bco2.70151

**Published:** 2026-02-04

**Authors:** Angelo Cormio, Daniele Castellani, Steffi Kar Kei Yuen, Khi Yung Fong, Deepak Ragoori, Laurian Dragos, Mohamed Omar, Luigi Cormio, Guohua Zeng, Wei Zhu, Shusheng Liu, Thomas R. W. Herrmann, Bhaskar K. Somani, Chi Fai Ng, Mohamed Elshazly, Vineet Gauhar

**Affiliations:** ^1^ Urology Unit Azienda Ospedaliero‐Universitaria delle Marche Ancona Italy; ^2^ Department of Urology and Renal Transplantation, Policlinico Riuniti di Foggia University of Foggia Foggia Italy; ^3^ Endourology Section of the European Association of Urology Arnhem the Netherlands; ^4^ Department of Surgery, S.H. Ho Urology Centre The Chinese University of Hong Kong Hong Kong China; ^5^ Department of Urology Sengkang General Hospital Singapore Singapore; ^6^ Department of Urology, Asian Institute of Nephrology & Urology Irram Manzil Colony Hyderabad Telangana India; ^7^ Urology Unit Cambridge University Hospitals NHS Foundation Trust Cambridge UK; ^8^ Urology Department Menoufia University Shibin el Kom Egypt; ^9^ Department of Urology and Andrology Bonomo Teaching Hospital Andria Italy; ^10^ Department of Urology and Guangdong Key Laboratory of Urology First Affiliated Hospital of Guangzhou Medical University Guangzhou China; ^11^ Department of Urology, Spital Thurgau AG Kantonsspital Frauenfeld Frauenfeld Switzerland; ^12^ Division of Urology, Department of Surgical Sciences Stellenbosch University Western Cape South Africa; ^13^ Hannover Medical School Hannover Germany; ^14^ Department of Urology University Hospital Southampton NHS Trust Southampton UK; ^15^ Department of Urology Ng Teng Fong General Hospital Singapore Singapore

**Keywords:** emphysematous pyelonephritis, infectious complications, kidney calculi, ureteral calculi, ureteroscopy, urinary tract infections

## Abstract

**Objective:**

This study aims to evaluate the safety, efficacy and clinical outcomes of elective flexible ureteroscopy using flexible and navigable suction ureteral access sheaths (FANS) in patients with upper tract infectious stones following initial management of systemic urinary tract infections (UTIs).

**Materials and Methods:**

We conducted a multicentre prospective analysis of patients with infectious ureteral or kidney stones (struvite or calcium carbonate‐apatite) who underwent flexible ureteroscopy using FANS between March 2024 and March 2025. All patients had prior systemic UTI management with a minimum 6‐week interval before definitive stone treatment. Stone‐free rate was assessed at 30 days using a CT scan.

**Results:**

The cohort included 144 patients (median age 51 years, 70.1% female) with a median stone diameter of 1.9 cm. 20.1% had initially received only antibiotic treatment, whereas 79.9% had also received emergency drainage. Forty‐seven patients (32.6%) had emphysematous pyelonephritis at initial presentation. Median operative time was 40 min with a 1‐day hospital stay. Notable findings included zero cases of sepsis and a 29.9% rate of postoperative fever requiring extended antibiotics up to 2 weeks. Zero residual fragments were achieved in 48.6% and a single fragment up to 2 mm in 43.8% of patients (92.4% combined stone‐free rate). Renal function improved at 30 days (median creatinine decrease −15 μmol/L) and 3 months (−18 μmol/L). Fever rate was 29.8% in the emphysematous pyelonephritis subgroup.

**Conclusions:**

Elective flexible ureteroscopy using FANS for upper tract infectious stones is feasible for its excellent safety profile with no sepsis cases and high stone‐free rates. FANS technology appears to offer significant advantages in this challenging patient population by mitigating postoperative sepsis due to debris aspiration, reduction of pyelovenous backflow of contaminated irrigation fluids and improved stone fragment evacuation.

## 1 INTRODUCTION

Kidney stone disease (KSD) is often associated with recurrent urinary tract infections (UTIs). Patients presenting with systemic UTIs (fever or hypothermia, hypotension, tachycardia and chills) represent a complex clinical challenge often needing urgent/emergent management due to upper tract obstructions, evolving into potentially life‐threatening complications such as sepsis and emphysematous pyelonephritis (EPN).^1^ Recurrent UTIs are commonly caused by infection‐related stones, namely, magnesium ammonium phosphate (struvite) or calcium carbonate‐apatite, which typically develop in alkaline urine environments created by urease‐producing bacteria.^2^, ^3^ Systemic UTIs require a staged approach, prioritizing immediate infection control with antibiotic therapy and urinary drainage before definitive stone removal. Following resolution of the acute infection episode, definitive stone management can be undertaken 4–6 weeks to ensure adequate infection control and patient optimization.^4^ Elective ureteroscopy is a safe procedure in patients who initially presented with sepsis with prior emergency drainage, provided that the infection is adequately treated.^5^ Pietropaolo et al. showed that only two out of 76 patients with prior urosepsis and emergency drainage had UTI requiring prolonged antibiotic therapy, and one patient developed sepsis.^5^ However, in another study, the incidence of postoperative fever in such patients was 9%.^6^ Moreover, performing ureteroscopy on infectious stones and those with UTI was found to have a 1.5% risk of intervention‐induced sepsis even in previously treated patients.^7^ Thus, retrograde intrarenal surgery (RIRS) in this subgroup of patients is a double‐edged sword. Surgeons have to ensure that to achieve the trifecta of RIRS (i.e., stone‐free, sepsis‐free and redo intervention‐free procedure),^8^ they do not potentiate the risk of repeat sepsis. As a consequence, ureteroscopic management of infectious stones presents unique technical challenges, including the need for comprehensive stone clearance to eliminate bacterial nidus, management of potentially purulent irrigation fluids due to ureteral stent/nephrostomy tube bacterial contamination and navigation in an inflamed urinary tract prone to injuries with the possibility of pyelovenous backflow‐induced infectious complications.^6^, ^9^

Suction in ureteroscopy using flexible and navigable suction ureteral access sheaths (FANS), a proven game changer in RIRS,^10^ offers theoretical advantages over standard sheaths for infectious stones in terms of complete stone clearance, improved irrigation flow dynamics^11^, ^12^, ^13^ and potentially lower risk of infectious complications.^14^ Nevertheless, the role of FANS in infectious stone management remains incompletely characterized, with limited data regarding safety and efficacy.

This study aimed to evaluate the feasibility, safety and outcomes of elective f‐URS using FANS in patients with upper tract infectious stones who had undergone initial emergency management for acute presentation of systemic UTIs.

## 2 METHODS

Data of patients presenting with systemic UTI and upper tract stones were prospectively collected from six centres (March 2024–March 2025). The study was approved by the Institutional Review Board of the leading centre (The Chinese University of Hong Kong, Hong Kong, China; CREC Reference Number: 2021.684), and other centres obtained approval from their institutional review board. All patients signed an informed consent form.

Inclusion criteria consisted of adult patients with infectious ureteral or kidney stones (i.e., struvite or calcium carbonate‐apatite) undergoing flexible ureteroscopy (f‐URS) with successfully deployed FANS after 6 weeks of a primary emergency treatment of systemic UTI with or without EPN.

Only those patients with either of the following clinical presentations and documented infectious‐related stones were eligible for inclusion:

1. Upper urinary tract obstruction due to stone(s) with culture‐positive urine
2. Clinical evidence of pyelonephritis or EPN, managed with appropriate antibiotics with or without upper tract drainage
3. Hospitalization for documented acute systemic UTI and diagnosed with incidental renal/ureteral stones diagnosed by computed tomography (CT) scan at this presentation.

All patients received antibiogram‐directed antibiotic therapy and upper urinary tract decompression with a double J ureteral stent or percutaneous nephrostomy tube, where deemed necessary.

Exclusion criteria encompass paediatric patients, f‐URS performed sheathless or with a standard sheath, and noninfectious stones. Patients with missing stone analysis were also excluded.

After initial antibiotic therapy, a repeat urine culture was done a week before f‐URS. If negative, antibiotic prophylaxis was given according to local practice. If still positive, patients had a week of antibiotics as per the antibiogram until surgery. A negative urine culture was not mandatory. Treatment regimens and antibiotic prophylaxis were administered according to local antibiogram results and institutional protocols at each participating centre, with all sites adhering to the fundamental principle of culture‐directed therapy and adequate infection control before elective intervention, in accordance with current EAU guidelines.^15^

Anticoagulants/antiplatelets were stopped 3–7 days before surgery and restarted according to each clinical condition. All patients had a preoperative CT scan at first presentation. Repeat CT scan before elective f‐URS with FANS was not mandatory.

The choice of energy source for lithotripsy, FANS, and perioperative decisions was at the respective surgeons' own discretion based on local available resources. Patients were assessed postoperatively according to the local standard of care. The following FANS was employed: ClearPetra System (Well Lead Medical Co Ltd, Guangzhou, China); Elephant II second‐generation FANS (Zhejiang YiGao Medical Technology Co., Ltd, Hangzhou, China) and FYS Ureteral Bendable Access Sheath (Hunan Reborn Medical Science and Technology Development Co., Ltd, Zhuzhou City, Hunan, China). The following disposable scopes were used: U‐Scope PRO (Zhuhai Pusen Medical Technology Co., Ltd, Guangdong, China); RP‐U‐C12 (RedPine Medical Equipment Co., Ltd., Guangdong, China); CoralView (MacroLux Medical Technology Co., Ltd., Guangdong Province, China); and Innovex Single‐Use 7.5 Fr Flexible Ureteroscope (Shanghai AnQing Medical Instrument Co., Ltd., Shanghai, China). An irrigation pump was employed in all cases.

At the end of each procedure, a 5‐point Likert‐type scale was used to assess surgeons' intraoperative experience, where 1 indicated *Excellent*, 2 *Very good*, 3 *Good*, 4 *Average* and 5 *Difficult*. Loin pain was evaluated on postoperative Day 1 using a 10‐point Visual Analog Scale, with 1 representing the lowest level of pain.

30‐day complications were reported using the modified Clavien‐Dindo classification system. Stone‐free rate (SFR) was evaluated 30 days after surgery using a postoperative 2 mm slice non‐contrast CT scan using bone window and graded as originally defined in the FANS registry^10^:

Grade A: zero residual fragments; 100% stone‐free;
Grade B: single fragment ≤2 mm;
Grade C: single residual fragment 2.1–4 mm;
Grade D: any stone >4 mm or multiple fragments of any size.

Patients with Grade C or D were planned for reintervention, and those with Grade A or B were considered stone‐free. All patients were followed up to 3 months with an X‐ray and ultrasound to look for any anatomical complications. Patients with EPN had a non‐contrast CT scan at 3 months to confirm complete resolution of the same.

### 2.1 Statistical analysis

Continuous variables are expressed as medians and interquartile ranges, while categorical variables are reported as absolute numbers and percentages. Variables and outcomes of interest for patients with EPN and ureteric stones were also reported. All statistical tests were performed using R version 4.1.2 (R Foundation for Statistical Computing, Vienna, Austria).

## 3 RESULTS

### 3.1 Overall population

A total of 144 patients were included. Table 1 shows baseline characteristics. Median age was 51.0 years (39.0–66.0), with a female predominance (70.1%). Most patients had an ASA score of 1 (58.3%). Comorbidities were common, with diabetes mellitus present in 30.6%, hypertension in 30.6% and ischemic heart disease in 33.3% of patients.

**TABLE 1:** Baseline and intraoperative characteristics of the overall series.

	*n* = 144
Age, years, median [IQR]	51.0 [39.0, 66.0]
Male gender, *n* (%)	43 (29.9)
ASA score, *n* (%)	
1	84 (58.3)
2	38 (26.4)
3	22 (15.3)
Body mass index, kg/m^2^, median [IQR]	26 [21, 30]
Diabetes mellitus, *n* (%)	44 (30.6)
Hypertension, *n* (%)	44 (30.6)
Ischemic heart disease, *n* (%)	48 (33.3)
Preoperative positive urine culture, *n* (%)	89 (61.8)
Emergency drainage at initial presentation, *n* (%)	
None	29 (20.1)
Double J ureteral stent	90 (62.5)
Nephrostomy tube	25 (17.4)
Right‐sided stone, *n* (%)	28 (19.4)
Stone largest diameter, cm, median [IQR]	1.9 [1.3, 2.5]
Hounsfield units, median [IQR]	1055.0 [938.0, 1222.0]
Guy's stone score, *n* (%)	
1	105 (72.9)
2	35 (24.3)
3	4 (2.8)
Stone location, *n* (%)	
Upper pole	19 (13.2)
Middle pole	46 (31.9)
Lower pole	23 (16.0)
Renal pelvis	37 (25.0)
Ureter	19 (13.2)
Normal kidney, *n* (%)	91 (63.2)
Anomalous kidneys, *n* (%)	
Duplex	6 (4.2)
Malrotated	29 (20.1)
Horseshoe kidney	18 (12.5)
Preoperative serum creatinine, umol/L, median [IQR]	116 [86, 150]

Abbreviations: ASA, American Society of Anesthesiologists; IQR, interquartile range.

Despite previous treatment, preoperative urine culture at the time of elective ureteroscopy was positive in 61.8% of patients. Emergency drainage at initial presentation was required in 79.9% of cases, with 62.5% receiving double J ureteral stents and 17.4% requiring a nephrostomy tube. Median largest stone diameter was 1.9 cm (1.3–2.5) with a median density of 1055 Hounsfield units (938–1222). Most stones were classified as Guy's stone score 1 (72.9%). Stone distribution showed middle calyceal location in 31.9%, renal pelvis in 25.0%, lower pole in 16.0%, upper pole in 13.2%, and ureteral location in 13.2% of cases. Anomalous kidneys were present in 36.8% of cases.

Table 2 shows operative characteristics. All procedures were performed under general anaesthesia. FANS sizes of 11–13 Fr were used in 52.8% of cases, while 10–12 Fr sheaths were employed in the rest. A 7.5 Fr scope was utilized in 87.1% of procedures, with 8.5 Fr scopes used in the remaining 22.9%. High‐power holmium laser was the most common energy source for lithotripsy (83.3%). Median ureteroscopy time was 20 min (10–30), while total operative time was 40 min (30–55). Of the 112 cases in whom intraoperative culture was taken, only seven were positive (4.9%). The majority of patients had a double J stent inserted at the end of the procedure (92.4%).

**TABLE 2:** Operative characteristics of the overall series.

	*n* = 144
General anaesthesia, *n* (%)	144 (100)
Sheath size, *n* (%)	
11–13 Fr	76 (52.8)
10–12 Fr	68 (47.2)
Scope size, *n* (%)	
7.5 Fr	111 (87.1)
8.5 Fr	33 (22.9)
Laser type, *n* (%)	
High‐power holmium laser	120 (83.3)
Low Power Holmium laser	5 (3.5)
Thulium fibre laser	6 (4.2)
Pulsed Thulium:YAG laser	13 (9.0)
Ureteroscopy time, min, median [IQR]	20 [10, 30]
Total operation time, min, median [IQR]	40 [30, 55]
Stone fragmentation, *n* (%)	
Dusting only	1 (0.7)
Combination of techniques	143 (99.3)
Stone basketing, *n* (%)	8 (5.6)
Sheath able to access all parts of the kidney, *n* (%)	144 (100)
Intraoperative culture, *n* (%)	
Not taken	112 (77.8)
Positive	7 (4.9)
Negative	25 (17.4)
Postoperative exit strategy, *n* (%)	
Double J stent	133 (92.4)
Overnight ureteric catheter	9 (6.2)
No stent or ureteric catheter	2 (1.4)
Likert scale rating for sheath performance*, *n* (%)	
Ease of suction	1 [1, 1]
Manipulation	1 [1, 2]
Visibility	3 [2, 4]

* only in kidney stone patients.

Table 3 reports postoperative outcomes. Minor bleeding during surgery occurred in 13.9% of patients, and blood transfusions due to multifactorial reasons (Clavien 2) were required in 2.1%. Grade 1 Thomas and Traxer ureteral injuries^16^ due to sheath placement and requiring prolonged stenting were documented in 5.6% of cases, affecting the ureteropelvic junction in 1.4% and the proximal ureter in 4.2%. Fever requiring 2 weeks of antibiotic therapy (Clavien 2) developed in 29.9% of patients. No cases of sepsis requiring intensive care admission were reported. Median Likert score was 1 for ease of manipulation and suction, while it was 3 for visibility. Median length of hospital stay was 1 day (1–2). Complete stone clearance (Grade A) was achieved in 48.6% of patients, while Grade B stone‐free status was attained in 43.8%. Only 5 (3.5%) patients required a reintervention.

**TABLE 3:** Postoperative outcomes of the overall series.

	*n* = 144
Minor bleeding, *n* (%)	20 (13.9)
Blood transfusion (Clavien 2), *n* (%)	3 (2.1)
Ureteric injury, *n* (%)	8 (5.6)
PUJ	2 (1.4)
Proximal ureter	6 (4.2)
PCS injury, *n* (%)	0
Fever requiring 2 weeks of antibiotics (Clavien 2), *n* (%)	43 (29.9)
Sepsis requiring intensive care admission, *n* (%)	0
Loin pain score, median [IQR]	2 [1, 2]
Length of hospital stay, days, median [IQR]	1 [1, 2]
Stone‐free rate	
Grade A	70 (48.6)
Grade B	63 (43.8)
Grade C	11 (7.6)
Planned for reintervention	5 (3.5)
Type of reintervention: ureterscopy	5 (3.5)
Change in serum creatinine from preoperatively, umol/L, median [IQR]	
24 h postoperative	0 [−26, 35]
30 days postoperative	−15 [−29, 0]
3 months postoperative	−18 [−35, −2.5]

Serum creatinine levels showed improvement from baseline over time. By 30 days, the median decrease was −15 μmol/L (−29 to 0), and at 3 months, the median reduction was −18 μmol/L (−35 to −2.5). No patient had a further episode of UTI within 3 months of surgery or died.

### 3.2 Subgroup analysis of EPN patients (Table S1)

Forty‐seven patients (32.6%) presented with Huang‐Tseng type 1 EPN.^16^ Median age of 40 years (36–67) with 27.7% male patients. Preoperative urine cultures were positive in 83.0% of cases. Emergency drainage was achieved with ureteral stents in 83.0% and nephrostomy tubes in 8.5%. EPN patients had larger stones with a median diameter of 2.0 cm (1.7–2.8). Fever requiring prolonged antibiotics (Clavien 2) occurred in 29.8% of patients. Median hospital stay was 2 days (1–2). SFRs were Grade A in 55.3% and Grade B in 27.7%. Renal function worsened at 24 h [median creatinine changes of +8.8 μmol/L (−18 to +35)], but it improved at 30 days [−8.8 μmol/L (−22 to +18)], and at 3 months [−18 μmol/L (−35 to 0)].

### 3.3 Subgroup analysis of ureteral stone patients (Table S2)

Nineteen patients (13.2%) presented with ureteral stones. Median age was 53 years (43–67.5) with 36.8% male patients. Emergency drainage was required in 26.4% (*n* = 5) of cases, with 21.1% receiving ureteral stents and 5.3% requiring nephrostomy tubes. Preoperative urine cultures were positive in only 21.1%. Ureteral stones were smaller, with a median diameter of 1.2 cm (1.0–1.9) and a density of 1062 HU (926–1430). Complications were infrequent in this subgroup, with fever requiring prolonged antibiotics (Clavien 2) in 5.3% and ureteral injury in 5.3%. Median hospital stay was 1 day (IQR 1–1). SFRs showed Grade A clearance in 43.1% and Grade B in 57.9%. Renal function improvement was more pronounced, with median creatinine decreases of −22 μmol/L (−31 to −0.4) at 24 h, −18 μmol/L (−36 to −5.0) at 30 days and −10 μmol/L (−39 to −4.0) at 3 months.

## 4 DISCUSSION

The interplay between KSD and UTIs remains incompletely understood, presenting a “chicken‐and‐egg” dilemma regarding which condition serves as the etiological factor and which arises as a consequence.^17^ UTIs play a well‐established etiopathogenetic role in the development of infection‐related calculi, specifically struvite and apatite stones. On the other hand, stones obstructing the upper urinary tract may cause systemic UTIs. In both cases, clinicians have to face complex clinical scenarios.

To the best of our knowledge, this is the first, largest prospective cohort specifically evaluating elective ureteroscopy with FANS focusing on patients with kidney or ureteral stone presenting with systemic UTIs, including challenging renal anatomies like anomalous kidneys and patients with EPN. Our findings demonstrate that elective f‐URS using FANS following initial emergency management of systemic UTIs represents a viable therapeutic approach with acceptable morbidity and encouraging SFRs in this challenging patient population. Crucially, this is probably the first series obtaining zero sepsis while achieving a very high SFR (Grade A + B = 92.3%), thus reiterating the debate that perhaps FANS should be used in all f‐URS cases.^9^ In literature too, FANS showed superior outcomes vis‐à‐vis traditional sheaths, and it is perhaps time to bury the no‐suction sheath.^18^

The most significant finding of our study remains the notable absence of sepsis, despite the high‐risk nature of our patients, contrasting favourably with historical data from conventional ureteroscopy in similar populations^5^, ^6^, ^19^ and supports the hypothesis that suction reduces intrarenal pressure (IRP) and therefore the risk of fluid absorption.^13^, ^14^ Moreover, it has been shown that the use of suction sheaths helps to keep operative times less than 60 min, and this is advocated as a significant contributor to reducing IRP.^7^ Indeed, in our cohort, the fact that FANS could reach different parts of the collecting system with ease, aspirate debris and multiple fragments at the same time is a practical and procedural benefit that helps achieve the excellent stone‐free and sepsis‐free results even in the presence of complex patients and with a median operative time of 40 minutes.

That said, our patients experienced a non‐negligible postoperative fever rate of 29.9%, which should, however, be evaluated in the context of infection stone management. Previous studies have reported fever rates of 6.6% to 10.8% following f‐URS, with a pooled rate of 2.6%,^20^ though these predominantly included noninfectious stones. In infectious stone patients, higher fever rates are anticipated due to the inherent bacterial burden, inflammatory milieu and higher risk of bacteraemia dissemination during lithotripsy.^13^, ^20^ A recent meta‐analysis examining f‐URS in suction technology in general urolithiasis populations demonstrated a 62% risk reduction in both postoperative infectious complications and fever and a 73% risk reduction in sepsis compared to conventional access sheaths,^21^ suggesting that the suction mechanism in FANS may mitigate infectious complications through improved fragment clearance and reduced IRP.

While percutaneous nephrolithotripsy is advocated as the best approach to manage infected systems by continuous drainage, a recent randomized study showed that FANS had noninferior SFR for 2–3‐cm renal stones compared with mini‐percutaneous nephrolithotripsy,^22^ with no difference in infectious complications despite similar rates of pure infection stones (6.6% vs. 5.3%). The authors suggested that f‐URS with FANS could be an equally good alternative. This corroborates the proposal that, as the landscape of endourological intervention evolves, suction might push the limits of f‐URS and evolve as a forerunner of a new era in expanding indications for its use.^22^

EPN patients represent a particularly challenging cohort, historically managed with PCNL or nephrectomy, with f‐URS often considered contraindicated due to worsening infection risks^23^, ^24^. In our series, EPN patients had a zero sepsis rate and a fever rate of 29.8%. While this is higher than other series of ureteroscopy for patients with ureteral stone and obstructive pyelonephritis,^25^ it compares well with most EPN series, whereby management of even Huang‐Tseng type 1 EPN was associated with a 4% risk of mortality, despite best intervention.^24^, ^26^ Our findings suggest that elective f‐URS using FANS may represent a viable minimally invasive alternative in appropriately selected patients with adequate initial drainage and infection control.

Of note, the technical success of FANS in our study was evidenced by the universal ability to access all parts of the collecting system. The excellent SFR can be considered another key factor in our study and likely contributed to the favourable outcomes of a low rate of infectious complications by minimizing residual fragments and associated bacterial nidus. The enhanced stone clearance observed in our cohort likely reflects the theoretical advantages of FANS technology, including improved irrigation dynamics and active fragment evacuation. The overall SFR (combining Grade A and B) achieved in our study compares favourably with published literature on ureteroscopy using FANS. A recent multicentre study examining FANS in the general kidney stone population has reported similar outcomes, demonstrating 30‐day Grades A + B SFRs of 97.2%,^10^ while comparative studies have shown 1.2‐fold higher final SFRs compared to conventional access sheaths.^21^

Another relevant outcome is the improved renal function at 30 days and 3 months post‐procedure, which likely reflects resolution of obstructive uropathy rather than direct procedural benefits. This pattern is consistent with previous studies demonstrating renal function recovery following relief of obstruction.^27^ The more pronounced improvement in the ureteral stone subgroup reflects the typically more acute obstructive picture in these patients.

An important consideration is that all procedures in our cohort were performed by experienced endourologists at tertiary referral centres, which may limit the generalizability of our findings to less experienced practitioners or lower volume centres. Proper training in FANS technology, including understanding of irrigation/suction dynamics and sheath manipulation techniques, is mandatory to achieve optimal outcomes, particularly when managing complex cases such as infectious stones. Yet, when using FANS, urologists should always adhere to the Quadrifecta of RIRS that relies on a careful balancing of suction, irrigation, temperature and pressure.^28^ The interplay among these factors necessitates ongoing adjustments to achieve optimal surgical outcomes and reduce the risk of complications.

This study has several limitations. First, the multicentre design introduces inherent bias regarding variability in surgical technique, and use of different types of FANS and lasers, as well as a lack of clarity on suction irrigation aspiration parameters but provides a good picture of real‐life data. Second, the 6‐week interval between initial presentation and definitive treatment, while clinically prudent, may have selected for patients with more favourable infection control, potentially improving outcomes. Again, delayed intervention after proper prior emergency and antibiotics management reflects real‐world and guideline‐based practice.^4^ The absence of severe complications, combined with high SFRs, supports the safety of this approach when performed in appropriate clinical settings with adequate patient selection and preparation. Third, the absence of a control group utilizing conventional access sheaths prevents direct comparison of outcomes; therefore, randomized controlled trials comparing FANS to conventional access sheaths in infectious stone patients are probably needed to definitively establish superiority. Fourth, stone volume was not calculated in our cohort, as we relied solely on largest diameter measurements for stone size assessment. This represents a further limitation, as three‐dimensional volumetric analysis may provide more accurate characterization of stone burden and potentially better correlation with outcomes such as operative time and SFR. Additionally, the lack of long‐term outcome data beyond 3 months precludes the ability to predict a reduction in stone recurrence rates and further UTIs. Finally, while we did not record IRP, enough evidence proved that FANS reduce the same.^11^, ^14^ Hence, FANS contributed to the favourable outcomes of a low rate of infectious complications by mitigating the bacteraemia and bacterial load and aspirating fragments and debris. This, combined with proper preoperative antibiotics, drainage and adequate postoperative antibiotic management strategy, allowed for a zero sepsis rate.

## 5 CONCLUSION

This study demonstrates that elective f‐URS using FANS for upper tract infectious stones is feasible, has acceptable safety profiles and excellent SFRs. The absence of severe infectious complications, combined with a low reintervention rate, suggests that FANS technology may represent a significant advancement in the management of this challenging patient population. While larger comparative studies are needed to definitively establish superiority over conventional sheaths, our findings support the continued investigation and clinical application of FANS in appropriately selected patients with infectious stone disease, but we reiterate that f‐URS with FANS is not for emergency management in infectious stones but is safely performed after adequate infection control. Moreover, future studies with longer follow‐up periods, particularly at 1 year, are warranted to evaluate late complications, stone recurrence rates and long‐term infectious outcomes in this challenging patient population.

## AUTHOR CONTRIBUTIONS

*Study conception*: Vineet Gauhar, Daniele Castellani and Mohamed Elshazly. *Data collection*: Mohamed Elshazly, Steffi Kar Kei Yuen, Khi Yung Fong, Deepak Ragoori, Laurian Dragos, Mohamed Omar, Guohua Zeng, Wei Zhu, Shusheng Liu. *Statistical analysis*: Khi Yung Fong and Daniele Castellani. *Manuscript writing*: Angelo Cormio, Daniele Castellani and Vineet Gauhar. *Manuscript review*: Luigi Cormio, Thomas R. W. Herrmann, and Bhaskar K. Somani. *Clinical supervision*: Chi Fai Ng and Guohua Zeng.

## CONFLICT OF INTEREST STATEMENT

Daniele Castellani certifies that all conflicts of interest, including specific financial interests and relationships and affiliations relevant to the subject matter or materials discussed in the manuscript (e.g., employment/affiliation, grants or funding, consultancies, honoraria, stock ownership or options, expert testimony, royalties or patents filed, received or pending), are the following: The authors declare no conflict of interest.

## Supporting information


**Table S1.** Subgroup analysis of emphysematous pyelonephritis patients.

**Table S2.** Subgroup analysis of ureteric stone patients.
