# Supplementary material for: Elective flexible ureteroscopy with suction sheaths for infectious stones in prior UTI patients
Source: BJUI Compass. 2026 Feb 4;7(2):e70151. doi: 10.1002/bco2.70151 (PMC12869839; doi:10.1002/bco2.70151)
Supplement: Supplementary file 1 — Table S1. Subgroup analysis of emphysematous pyelonephritis patients. [file BCO2-7-e70151-s001.docx]

# Supplementary Table 1. Subgroup analysis of emphysematous pyelonephritis patients.

|  | n=47 |
| --- | --- |
| Age, years, median [IQR] | 40 [36, 67] |
| Male gender, n (%) | 13 (27.7) |
| Preoperative positive urine culture, n (%) | 39 (83.0) |
| Emergency drainage at initial presentation, n (%)  None  Double J ureteral stent  Nephrostomy tube, | 4 (8.5)  39 (83.0)  4 (8.5) |
| Stone largest diameter, cm, median [IQR] | 2.0 [1.7, 2.8] |
| Hounsfield units, median [IQR] | 1100 [1010, 1261] |
| Guy’s stone score  1  2  3 | 30 (63.8)  15 (31.9)  2 (4.3) |
| Stone location  Upper pole  Middle pole  Lower pole  Renal pelvis  Ureteropelvic junction  Ureter | 3 (6.4)  16 (34.0)  11 (23.4)  5 (10.6)  11 (23.4)  1 (2.1) |
| Normal kidney, n (%) | 26 (55.3) |
| Preoperative serum creatinine, umol/L, median [IQR] | 115 [71, 159] |
| Length of hospital stay, days, median [IQR] | 2 [1, 2] |
| Stone-free rate, n (%)  Grade A  Grade B  Grade C | 26 (55.3)  13 (27.7)  8 (17.0) |
| Fever requiring 2 weeks of antibiotics (Clavien 2), n (%) | 14 (29.8) |
| Ureteric injury requiring prolonged stent, n (%) | 2 (4.3) |
| Reintervention (ureteroscopy), n (%) | 1 (2.1) |
| Change in serum creatinine from preoperatively, umol/L, median [IQR]  24 hours postoperative  30 days postoperative  3 months postoperative | 8.8 [-18, 35]  -8.8 [-22, 18]  -18 [-35, 0] |
