# Supplementary material for: Elective flexible ureteroscopy with suction sheaths for infectious stones in prior UTI patients
Source: BJUI Compass. 2026 Feb 4;7(2):e70151. doi: 10.1002/bco2.70151 (PMC12869839; doi:10.1002/bco2.70151)
Supplement: Supplementary file 2 — Table S2. Subgroup analysis of ureteric stone patients. [file BCO2-7-e70151-s002.docx]

**Supplementary Table 2.** Subgroup analysis of ureteric stone patients.

|  | n=19 |
| --- | --- |
| Age, years, median [IQR] | 53 [43, 67.5] |
| Male gender, n (%) | 7 (36.8) |
| Preoperative positive urine culture, n (%) | 4 (21.1) |
| Emergency drainage at initial presentation, n (%)  None  Double J ureteral stent  Nephrostomy tube | 14 (73.6)  4 (21.1)  1 (5.3) |
| Stone largest diameter, cm, median [IQR] | 1.2 [1.0, 1.9] |
| Hounsfield units, n (%) | 1062 [926, 1430] |
| Normal kidney, n (%) | 19 (100) |
| Preoperative serum creatinine, umol/L, median [IQR] | 102 [85, 135] |
| Length of hospital stay, days, median [IQR] | 1 [1, 1] |
| Stone-free rate, n (%)  Grade A  Grade B  Grade C | 8 (43.1)  11 (57.9)  0 |
| Fever requiring 2 weeks of antibiotics (Clavien 2), n (%), n (%) | 1 (5.3) |
| Ureteric injury requiring prolonged stenting, n (%) | 1 (5.3) |
| Reintervention (ureteroscopy), n (%) | 1 (5.3) |
| Change in serum creatinine from preoperatively, umol/L  24 hours postoperative  30 days postoperative  3 months postoperative | -22 [-31, -0.4]  -18 [-36, -5.0]  -10 [-39, -4.0] |
